# Biochar stimulates tomato roots to recruit a bacterial assemblage contributing to disease resistance against *Fusarium* wilt

**DOI:** 10.1002/imt2.37

**Published:** 2022-06-23

**Authors:** Xue Jin, Yang Bai, Muhammad Khashi u Rahman, Xiaojun Kang, Kai Pan, Fengzhi Wu, Thomas Pommier, Xingang Zhou, Zhong Wei

**Affiliations:** ^1^ Key Laboratory of Biology and Genetic Improvement of Horticultural Crops (Northeast Region), Ministry of Agriculture and Rural Affairs, Department of Horticulture Northeast Agricultural University Harbin China; ^2^ Department of Plant & Microbial Biology University of Minnesota Saint Paul Minnesota USA; ^3^ Univ Lyon, Université Claude Bernard Lyon 1, CNRS, INRAE, VetAgro Sup, UMR Ecologie Microbienne Villeurbanne France; ^4^ Jiangsu Provincial Key Lab for Organic Solid Waste Utilization, Laboratory of Bio‐interactions and Crop Health, National Engineering Research Center for Organic‐based Fertilizers, Jiangsu Collaborative Innovation Center for Solid Organic Waste Resource Utilization Nanjing Agricultural University Nanjing China

**Keywords:** bacterial diversity, disease suppression, *Fusarium* wilt, microbiome recruitment

## Abstract

Biochar amendment is acknowledged to favor plant resistance against soil‐borne diseases. Although plant‐beneficial bacteria enrichment in the rhizosphere is often proposed to be associated with this protection, the mechanism behind this stimulating effect remains unelucidated. Here, we tested whether biochar promotes plants to recruit beneficial bacteria to the rhizosphere, and thus develop a disease‐suppressive rhizosphere microbiome. In a pot experiment, biochar amendment decreased tomato *Fusarium* wilt disease severity. Using a transplanting rhizosphere microbiome experiment, we showed that biochar enhanced the suppressiveness of tomato rhizosphere microbiome against *Fusarium* wilt disease. High‐throughput sequencing of 16S ribosomal RNA gene and in vitro cultures further indicated that the recruited suppressive rhizosphere microbiome was associated with the increase of plant‐beneficial bacteria, such as *Pseudomonas* sp. This amendment also enhanced the in vitro chemoattraction and biofilm promotion activity of tomato root exudates. Collectively, our results demonstrate that biochar amendment induces tomato seedlings to efficiently recruit a disease‐suppressive rhizosphere microbiome against *Fusarium* wilt.

## Highlights

- Biochar enhances the disease suppressiveness of tomato rhizosphere microbiome.
- Biochar stimulates tomato to actively recruit plant‐beneficial bacterial taxa.
- The ability of tomato root exudates to recruit beneficial bacteria is enhanced by biochar.

## Graphical abstract

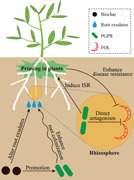

## INTRODUCTION

Plant diseases cause serious economic losses in agriculture, and thus threaten the global food security [1]. Particularly, tomato (*Solanum lycopersicum* L.) *Fusarium* wilt disease, caused by the soil‐borne necrotrophic fungus *Fusarium oxysporum* f. sp. *lycopersici* (FOL), is one of the major yield‐limiting factors in tomato production [2, 3]. Soil‐borne diseases are often difficult to control with conventional strategies such as the use of resistant host cultivars and synthetic fungicides [4, 5]. Recently, using biochar as a soil amendment has been suggested as an alternative agricultural management practice to control plant diseases, such as plant soil‐borne diseases caused by pathogens such as *Fusarium* spp., *Pythium* spp., and *Rhizoctonia solani* [6–10]. Biochar is a solid, carbon‐rich product derived from the pyrolysis of organic materials [10–13]. To date, several mechanisms have been proposed to explain the biochar‐related attenuation of soil‐borne diseases, such as changes in soil physicochemical characteristics (e.g., soil pH), improved nutrient supply to plants, induction of systematic plant defenses, and reduced pathogen abundance and virulence [6, 8, 9, 14–16]. Recent studies indicate that biochar‐induced plant disease suppression may be linked to the changes in the rhizosphere microbiome [17–19], but the underlying mechanism remains largely unclear.

Plants interact intimately with both symbiotic mutualists and pathogens in their rhizosphere [20, 21]. Plant rhizosphere microbiome is a key determinant of plant health and productivity, and is considered as a major driver of plant defense against belowground pathogens [22–24]. Plant growth‐promoting rhizobacteria (PGPR) can confer resistance against plant pathogens by (i) direct antagonism (e.g., parasitism, antibiosis, or competition for resources or infection sites) or (ii) via the host plants by triggering induced resistance [25]. A growing body of evidence demonstrates that biochar amendment alters the composition and diversity of microbial communities, and stimulates the abundance of PGPRs in the rhizosphere [17–19, 26, 27]. Generally, such positive effects conferred to PGPRs have mainly been attributed to the inherent properties of biochar and changes in soil physicochemical properties induced by biochar [28, 29].

Plants secrete a plethora of chemically diverse low molecular weight compounds (i.e., root exudates) into the rhizosphere [30]. Root exudates, that act as substrates and signaling molecules for rhizosphere microorganisms, govern numerous rhizosphere processes including the recruitment of PGPR and shaping of the rhizosphere microbiome [21, 31–34]. The quantity and composition of root exudates depend on the plant genotype, developmental stage, and growth conditions (e.g., nutrient availability) [30, 34]. Recent evidence shows that biochar amendment could alter the chemistry of plant root exudates [2, 35, 36]. Akhter et al. [2] found that root exudates from tomato plants treated with biochar had an inhibitory effect on in vitro growth of FOL compared with that from tomato plants untreated with biochar. Therefore, it is possible that biochar amendment can increase the abundance of PGPR in plant rhizosphere through the host plant.

Here, we hypothesized that biochar amendment could stimulate tomato seedlings to efficiently recruit a disease‐suppressive rhizosphere microbiome against *Fusarium* wilt. To test this hypothesis, we (i) tested the effects of biochar amendment on tomato seedling growth and *Fusarium* disease severity; (ii) investigated the role of the rhizosphere microbiome in suppressing *Fusarium* disease severity by biochar; (iii) evaluated the changes in rhizosphere bacterial communities induced by biochar, and isolated and characterized the bacteria that were stimulated by biochar; and (iv) tested whether biochar stimulated tomato to actively recruit PGPR.

## RESULTS

### Biochar amendment enhanced tomato seedling performance

**Figure 1:**
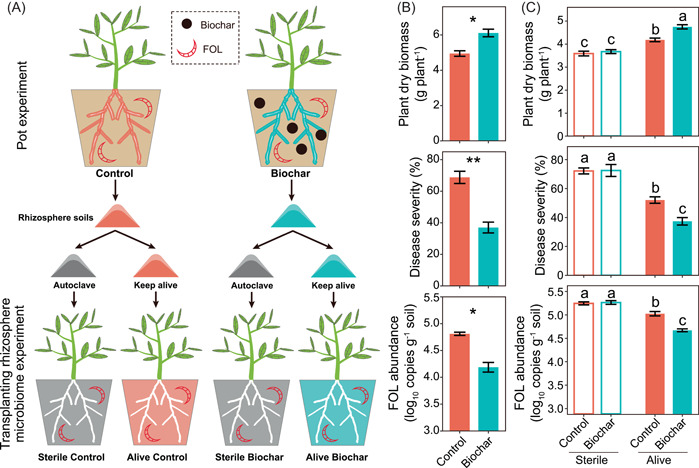
Schematic representation of the pot and transplanting rhizosphere microbiome experiments and tomato seedling performance. (A) Schematic representation of the experiments. In the pot experiment, tomato seedlings were grown in soils not amended with biochar (Control), or amended with 1% biochar (Biochar), respectively. For the transplanting rhizosphere microbiome experiment, tomato seedlings were grown in sterilized field soils mixed with sterile or nonsterile rhizosphere soils from the pot experiment, respectively. Tomato seedling dry biomass and *Fusarium* wilt disease severity in the pot experiment (B) and the transplanting rhizosphere microbiome experiment (C). * and ** indicate significant difference at *p* < 0.05 and *p* < 0.01 (Welch's *t *test), respectively. Different letters indicate significant differences (Tukey's honestly significant difference test, *p* < 0.05). FOL, *Fusarium oxysporum* f. sp. *lycopersici*.

In the pot experiment (Figure 1A), the biochar treatment increased tomato seedling dry biomass by 23.52% as compared to the nonamended control (Welch's *t *test, *p* < 0.05; Figure 1B). Moreover, the biochar treatment decreased tomato seedling *Fusarium* wilt disease index (by 46.17%) and FOL abundance in tomato rhizosphere (by 12.99%) as compared with the nonamended control (Welch's *t *test, *p* < 0.05; Figure 1B).

### Rhizosphere microbiome contributed to the enhanced resistance against *Fusarium* wilt disease

In the transplanting rhizosphere microbiome experiment (Figure 1A), tomato seedlings' dry biomass, *Fusarium* wilt disease index and rhizosphere FOL abundance did not differ among treatments inoculated with sterile rhizosphere soils of tomato seedlings grown in soils amended or that nonamended with biochar (i.e., Sterile Control vs. Sterile Biochar) (Figure 1C). However, as compared with the treatment inoculated with nonsterile rhizosphere soils of tomato seedlings grown in soils nonamended with biochar (i.e., Nonsterile Control), the treatment inoculated with nonsterile rhizosphere soils of tomato seedlings grown in soils amended with biochar (i.e., Nonsterile Biochar) increased tomato seedlings dry biomass (by 13.55%), while decreased *Fusarium* wilt disease index (by 28.24%) and rhizosphere FOL abundance (by 7.04%) (Tukey's honestly significant difference [HSD] test, *p* < 0.05).

### Biochar altered tomato rhizosphere bacterial diversity and community composition

**Figure 2:**
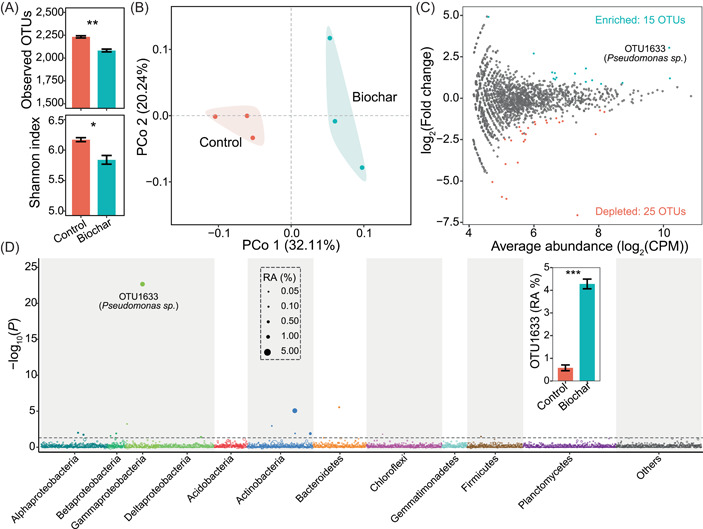
Diversity and composition of tomato rhizosphere bacterial community in the pot experiment. (A) The α‐diversity of tomato rhizosphere bacterial community by observed OTUs and Shannon index. (B) PCoA of tomato rhizosphere bacterial community β‐diversity. (C) Volcano plot showing OTUs enriched or depleted in the biochar treatment as compared with the control (Likelihood ratio test, Benjamini–Hochberg corrected *p* < 0.05). (D) The Manhattan plot shows the taxonomic information of OTUs enriched in the biochar treatment. The dashed line represents the threshold of significance (Benjamini–Hochberg corrected *p* = 0.05). The inset bar plot shows the RA of OTU1633. Tomato seedlings were grown in soils not amended with biochar (Control) or amended with 1% biochar (Biochar), respectively. *, **, and *** indicate significant difference at *p* < 0.05, *p* < 0.01, and *p* < 0.001 (Welch's *t *test), respectively. CPM, count per million; OTU, operational taxonomic units; PCoA, principal coordinate analyses; RA, relative abundance.

Illumina‐based 16 ribosomal RNA (rRNA) gene amplicon sequencing generated a total of 234,125 reads with an average of 39,020 reads per sample, from which 219,116 high‐quality reads (36,519 on average per sample) were selected for downstream analysis. Rarefaction curves of operational taxonomic units (OTUs) at 97% sequence similarity of all samples tended to approach the saturation plateau (Supporting Information: Figure S1). The average Good's coverage for all samples was 98.00 ± 0.03% (±SE), indicating that the sampling was adequate. Biochar amendment significantly decreased the number of OTUs and Shannon index of tomato seedling rhizosphere bacterial community (Welch's *t *test, *p* < 0.05; Figure 2A). Principal coordinates analysis (PCoA), based on the Bray–Curtis dissimilarities, showed that bacterial communities of the biochar treatment were distinctly different from those of the nonamended control (Figure 2B).

The vast majority of rhizosphere bacteria in the biochar treatment and nonamended control [88.43 ± 0.65% and 90.78 ± 0.44% (±SE), respectively] belonged to six dominant bacterial phyla (relative abundance > 5%): *Proteobacteria*, *Actinobacteria*, *Firmicutes*, *Acidobacteria*, *Bacteroidetes*, and *Chloroflexi*. The relative abundance of bacterial class *Gammaproteobacteria* was significantly higher, while that of class *Deltaproteobacteria*, phyla Acidobacteria and *Chloroflexi*, in the biochar treatment were lower than in the nonamended control (Welch's *t *test, *p* < 0.05; Supporting Information: Figure S2a). The likelihood ratio test found that biochar amendment increased and decreased the relative abundances of 15 and 25 OTUs, respectively, as compared with the nonamended control (Figure 2C). These stimulated OTUs mainly belonged to Proteobacteria (seven OTUs) and Actinobacteria (four OTUs) (Supporting Information: Figure S2b). Particularly, biochar amendment stimulated the OTU1633, belonging to *Pseudomonas* sp. (Welch's *t *test, *p* < 0.05; Figure 2D). The relative abundance of all *Pseudomonas* sp. was also stimulated by biochar amendment (Supporting Information: Figure S2c). Quantitative PCR analysis found that biochar amendment increased the abundance of *Pseudomonas* sp. in tomato rhizosphere (Welch's *t *test, *p* < 0.05; Supporting Information: Figure S2c).

### Isolated *Pseudomonas* sp. suppressed tomato *Fusarium* wilt disease

**Figure 3:**
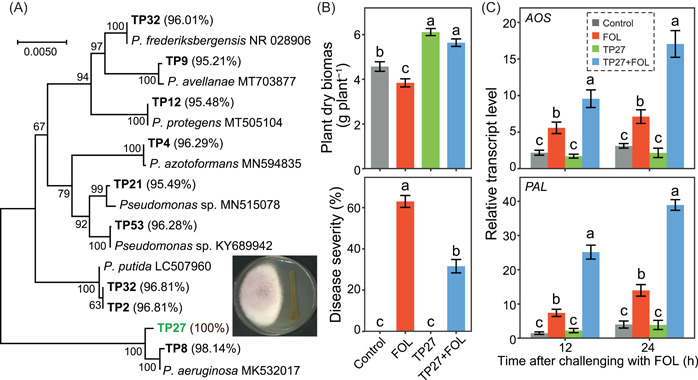
Isolated *Pseudomonas* sp. and the effects of TP27 on tomato seedling growth and disease resistance. (A) The neighbor‐joining tree shows the phylogenetic relationships of isolated *Pseudomonas* sp. Isolates from this study are in bold letters. Numbers within parentheses are the sequence similarities of each isolate with OTU1633. Reference strains in the NCBI database with their accession numbers are in regular letters. Bootstrap values are based on 1000 resampling and shown at the branching points. Effects of *Pseudomonas* TP27 on tomato seedling dry biomass, *Fusarium* wilt disease severity (B) and defense‐related gene expressions (C). Tomato seedlings were grown in soils challenged with FOL, inoculated with TP27 (TP27), or both inoculated with TP27 and challenged with FOL (TP27+ FOL). The Control treatment was treated with water. Different letters indicate significant differences (Tukey's HSD test, *p* < 0.05). FOL, *Fusarium oxysporum* f. sp. *lycopersici*; HSD, honestly significant difference.

We were tempted to isolate culturable *Pseudomonas* sp. in tomato rhizosphere and then test its effects on tomato seedling growth and resistance to *Fusarium* wilt disease. We selected the isolate TP27 from 10 *Pseudomonas* sp. isolates because it displayed 100% sequence similarity with OTU1633 (Figure 3A). We found that TP27 showed in vitro antagonistic activity against FOL on potato–dextrose–agar (PDA) agar (imbedded picture in Figure 3A). When inoculated into the soil, TP27 promoted tomato seedling biomass by 33.73% and 46.88% when noninoculated and inoculated with FOL, respectively (Tukey's HSD test, *p* < 0.05; Figure 3B). TP27 also decreased *Fusarium* wilt disease index by 49.87% when inoculated with FOL (Tukey's HSD test, *p* < 0.05; Figure 3B). Compared with the uninoculated control, inoculation of TP27 alone did not alter the expression levels of defense‐related genes (i.e., genes encoding allene oxide synthase (*AOS*), phenylalanine ammonia‐lyase (*PAL*) that are involved in jasmonic acid and salicylic acid signaling pathways, respectively) in tomato seedling roots, while inoculation of the pathogen FOL alone resulted in increases in the expressions of *AOS* and *PAL* in tomato seedling roots (Tukey's HSD test, *p* < 0.05; Figure 3C). However, inoculation of the pathogen on seedlings preinoculated with TP27 resulted in enhanced upregulation of FOL‐induced *AOS* and *PAL* expressions in tomato roots (Tukey's HSD test, *p* < 0.05). Thus, suppression of tomato *Fusarium* wilt by TP27 was accompanied by primed expressions of these defence‐related genes.

### Biochar promoted *Pseudomonas* sp. TP27 in the absence of plant

To test whether biochar amendment stimulates PGPR in the absence of plants, a microcosm experiment in sterile soils inoculated with TP27 was performed. Biochar amendment increased TP27 population density by 1.19%, 2.17%, and 2.78% on 7, 14, and 21 days after treatment, respectively, as compared with the nonamended control (Welch's *t *test, *p* < 0.05; Supporting Information: Figure S3).

### Biochar stimulated the colonization of *Pseudomonas* sp. TP27 on tomato root

**Figure 4:**
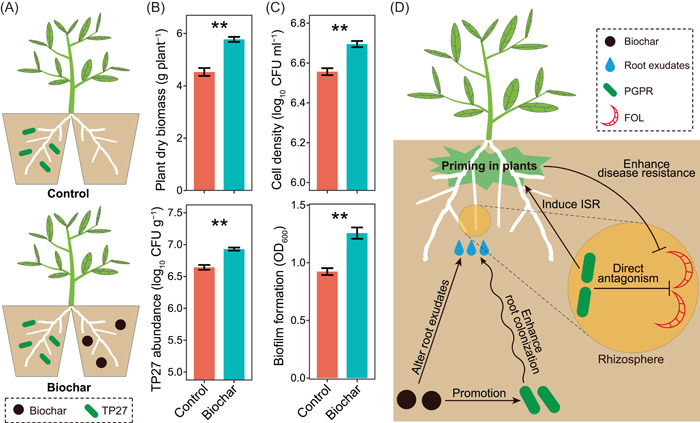
*Pseudomonas* sp. TP27 colonization on tomato roots and the conceptual model of our results. (A) Schematic representation of the split‐root system experiment. One part of the tomato root system was inoculated with TP27, and the other part was untreated (Control) or amended with biochar (Biochar), respectively. (B) Tomato seedling dry biomass and rhizosphere TP27 abundance in the split‐root system experiment. (C) In vitro effects of tomato seedling root exudates on the chemotaxis and biofilm formation of TP27. ** indicates significant differences at *p* < 0.01 (Welch's *t *test). (D) Conceptual model showing how biochar amendment suppresses tomato *Fusarium* wilt disease through altering the rhizosphere microbiome. CFU, colony‐forming unit; FOl, *Fusarium oxysporum* f. sp. *lycopersici*; OD, optical density; PGPR, plant growth‐promoting rhizobacteria.

To test whether biochar amendment stimulates PGPR in tomato rhizosphere via the host plant, a split‐root system of tomato was used (Figure 4A). As compared with the nonamended control, biochar amendment significantly promoted tomato seedling growth (by 27.52%) and increased the abundance of TP27 in the rhizosphere of roots grown in soil inoculated with TP2 (by 4.32%) (Welch's *t *test, *p* < 0.05; Figure 4B).

We also tested the role of tomato root exudates in this enhanced colonization of tomato roots by TP27. In vitro tests showed that root exudates of tomato grown in soil inoculated with TP2 from the biochar treatment promoted the chemotaxis response (by 2.11%) and biofilm formation of TP27 (by 36.10%) as compared with that from the nonamended control (Welch's *t *test, *p* < 0.05; Figure 4C).

## DISCUSSION

The positive effects of biochar amendment on crop production, especially for the suppression of plant diseases, have been well documented [6, 7, 10]. The plant rhizosphere microbiome is a key determinant of plant health [23, 24, 37]. Although numerous studies found that plant rhizosphere microbiome could be altered by biochar amendment [17–19, 26, 27], evidence proving a direct link between biochar‐induced changes in the rhizosphere microbiome and effective disease suppression is still rare [6]. Here, our transplanting rhizosphere microbiome experiment showed that the rhizosphere microbiome from tomato treated with biochar had a higher ability to decrease tomato *Fusarium* wilt disease severity than that from untreated with biochar. Thus, changes in the rhizosphere microbiome induced by biochar contributed to the disease‐suppressing ability of biochar amendment.

In this study, the suppression of *Fusarium* wilt disease induced by biochar was associated with the enrichment of specific bacteria taxa, especially *Pseudomonas* sp., in tomato rhizosphere. Members in *Pseudomonas* sp. are widely used as biocontrol agents to suppress soil‐borne plant diseases, including tomato *Fusarium* wilt disease [5, 38, 39]. This protective effect has been linked to several mechanisms, such as direct antagonistic activity against the pathogen and triggering induced resistance in the host plants [25]. The latter typically relies on jasmonic acid/ethylene‐dependent signaling pathways and can also be triggered via salicylic acid‐dependent signaling pathways [25, 40]. *AOS* and *PAL* are important for the synthesis of the defense hormone jasmonic acid and salicylic acid, respectively [41, 42]. The expressions of *AOS* and *PAL* following pathogen attack were boosted in tomato seedling roots by *Pseudomonas* sp. TP27, indicating this bacterium, exerted a priming effect on defense‐related gene expression, that is, a faster and stronger expression of defense responses that become activated upon pathogen attack [25]. Recent studies showed that biochar amendment induced a primed state in plants against pathogens, which was similar to that primed by PGPR [9, 15, 43]. Mehari et al. [9] found that biochar‐mediated induced systemic resistance to *Botrytis cinerea* in tomato involved the jasmonic acid signaling. Therefore, the stimulated PGPR in the rhizosphere is one of the potential elicitors of such biochar‐mediated priming for systemic resistance.

Our microcosm experiment showed that biochar stimulated *Pseudomonas* sp. TP27 in the absence of the host plant, which indicated that the increased PGPR abundance might be due to the direct effect of biochar on PGPR or via the altered soil physicochemical as previously reported [28, 29]. The colonization of PGPR on plant roots is a process that can be actively regulated by the plant [24, 30]. Results from our split‐root system experiment provided direct evidence for the concept that biochar could stimulate tomato seedlings to actively recruit specific PGPR groups (e.g., *Pseudomonas* sp.), which validated our hypothesis. Thus, our results highlighted the importance of the host plants in the biochar‐induced development of the disease‐suppressive rhizosphere microbiome. Especially, biochar amendment altered plant's physiological status, especially the root exudation, which contributed to the recruitment of PGPR. Previous studies showed that the biochar can directly affect the plant growth and physiological status [44, 45], and indirectly by altering soil physiochemical characteristics, such as soil pH, soil moisture, and nutrient availability [7]. Future investigations would be necessary to test these potential mechanisms underlying how biochar alters plant's traits that are involved in the recruitment of PGPR.

Plant root exudates are an important source of nutrients and signaling molecules for rhizosphere microbial communities, and play an important role in the recruitment of PGPR [30, 46]. Chemotaxis and biofilm formation are key processes during the rhizosphere colonization of PGPR [47]. PGPR use chemotaxis to sense and respond to plant‐derived signals in plant exudates and to initiate colonization. Subsequently, microorganisms move toward the plant rhizosphere, and then attach to the root surface and form a biofilm [24]. Our in vitro chemotaxis assays revealed an increased chemotaxis of *Pseudomonas* sp. TP27 toward root exudates of tomato seedlings treated with biochar compared with that of the untreated seedlings. Moreover, biochar amendment enhanced the ability of tomato root exudates to stimulate biofilm formation of *Pseudomonas* sp. TP27. These results indicated that changes in root exudates were involved in the biochar‐stimulated recruitment of PGPR in tomato rhizosphere. However, we still do not know that biochar stimulated which specific compound(s) in tomato root exudates to recruit *Pseudomonas* sp. TP27 and reshaped the rhizosphere microbiome. Therefore, future studies analyzing the composition of tomato root exudates may improve our understanding of the detailed role of the plant in the biochar‐induced building up of soil suppressiveness.

## CONCLUSIONS

This study showed that biochar‐elicited recruitment of PGPR contributed to the enhanced disease suppressiveness of tomato rhizosphere microbiome against *Fusarium* wilt (Figure 4D). Our results highlighted the importance of the host plant in this enhanced recruitment of PGPR. Biochar amendment altered the root exudates of tomato, and thus stimulated tomato seedling to selectively recruit specific PGPR (e.g., *Pseudomonas* sp.) to tomato rhizosphere. The enriched PGPR in the rhizosphere could protect tomato through direct antagonistic activity and induction of systemic resistance in the plant. Our results also suggest that enhancing the recruitment of beneficial microbial communities in the rhizosphere through agricultural practices can help to suppress soil‐borne diseases, and thus increase agricultural sustainability.

## METHODS

### Soil, biochar, and FOL conidia preparation

A cropland soil sample (0–20 cm depth) was collected from the Xiangyang village, Harbin, China (45°78′ N, 126°94′ E). The soil sample was sieved (<2mm) and analyzed for physicochemical properties with the following values: 48.32 g kg^−1^ of organic matter, 67.55 mg kg^−1^ of inorganic nitrogen, 186.57 mg kg^−1^ of available phosphorus, 190.37 mg kg^−1^ of available potassium, electrical conductivity (1:2.5, w/v) of 0.34 mS cm^−1^; pH (1:2.5, w/v) of 7.01. The biochar was prepared from Jerusalem artichoke (*Helianthus tuberosus* L.) stalks by pyrolysis at 450°C for 4 h in an N_2_ atmosphere (N_2_ flow rate, 100 ml min^−1^). The main characteristics of the biochar were: specific surface area 2.58 m^2^ g^−1^, cation exchange capacity 8.79 cmol_c _kg^−1^, pH 9.24, ash content 38.52%, 56.78 mg kg^−1^ of inorganic nitrogen, 139.23 mg kg^−1^ of available phosphorus, and 4.15 g kg^−1^ of available potassium.

Isolate FOL06 (race 1) of FOL was used in this study. This isolate was maintained on PDA medium and its conidia were obtained as described before [3].

### Pot experiment

A pot experiment was performed to evaluate the effect of biochar amendment on tomato seedling growth and *Fusarium* wilt disease severity (Figure 1A). There were two treatments: (i) soil not amended with biochar (Control), and (ii) soil amended with biochar at the rate of 1% (w/w) (Biochar). After mixing thoroughly, all soils were incubated for 2 weeks with soil water content maintained at about 60% of water holding capacity (WHC) at room temperature [18]. Then, tomato seedlings (cultivar “DN702,” susceptible to FOL06) with two leaves were transplanted into plastic pots (14 cm in diameter, 16 cm in height) containing 1.0 kg of different soils. There was one seedling per pot. Each treatment was replicated three times, and each replicate contained 35 pots. All pots were placed randomly in the greenhouse (average day/night temperature 32°C/22°C, relative humidity 60%–80%, 16 h light), and the position was randomly changed every 3 days. Soil water content was adjusted every 2 days with distilled water to maintain the soil moisture at about 60% of WHC.

Thirty days after transplantation, 20 tomato seedlings of each treatment in each replicate were harvested to measure dry biomass and collect rhizosphere soils (Supporting Information: Methods). One portion of these freshly sampled soils was used for the transplanting rhizosphere microbiome experiment and isolating culturable bacteria, and the other portion was stored at −80°C for rhizosphere microbiome analysis. Tomato seedling biomass was determined after oven drying at 70°C to a constant weight.

Meanwhile, 15 seedlings of each treatment in each replicate were challenged with FOL by directly pipetting 10 ml of conidial suspension of FOL06 (1.0 × 10^7^ conidia ml^−1^) onto the soil surface of each pot. Fifteen days later, the percentage of leaf yellowing/wilting was used to evaluate disease severity and was calculated using a scale containing nine grades [3].

### Transplanting rhizosphere microbiome experiment

A transplanting rhizosphere microbiome experiment was used to evaluate the role of the rhizosphere microbiome in mediating the suppression of *Fusarium* wilt by biochar amendment. The method of adding soil inoculum to sterilized background soils was used as previously described [48, 49] (Figure 1A). Briefly, cropland soils were sterilized by two cycles of autoclaving (121°C, 20 min) and cooling to room temperature, and were used as the background soil. Tomato rhizosphere soils from the pot experiment were used as inocula. Half of these inocula were sterilized, while the other half were kept alive. The absence of culturable microbes in autoclaved soils was confirmed by plating soil dilutions on Luria–Bertani (LB) agar medium. There were four treatments: sterilized background soils mixed with (i) sterile tomato rhizosphere soils of the control (Sterile Control), (ii) sterile tomato rhizosphere soils of the biochar treatment (Sterile Biochar), (iii) nonsterile tomato rhizosphere soils of the control (Nonsterile Control), and (iv) nonsterile tomato rhizosphere soils of the biochar treatment (Nonsterile Biochar). The ratio of inoculum‐to‐background soil was 6% (w/w). Sterilized background soils were mixed with each inoculum in sterile flasks by shaking. Three days later [49], tomato seedlings with two leaves were transplanted into plastic pots (14 cm in diameter, 16 cm in height) containing 1.0 kg of these soil mixtures. There was one seedling per pot. Each treatment was replicated three times, and each replicate contained five pots. Seedlings were managed as described above. Fifteen days after transplantation, tomato seedlings were challenged with FOL. Another 15 days later, tomato seedling biomass and *Fusarium* wilt disease severity were measured as described above.

### DNA extraction, amplicon sequencing, and quantitative PCR analyses

Genomic DNA was extracted from 0.25 g of tomato rhizosphere soils from the pot experiment with the OMEGA‐soil DNA Kit (Omega Bio‐Tek) following the manufacturer's protocol. The quality of the extracted DNA was checked on 1.2% (w/v) agarose gel and a NanoDrop 2000 spectrophotometer (Thermo Fisher Scientific).

Tomato rhizosphere bacterial diversity and community composition were analyzed with amplicon sequencing. The V4–V5 regions of the bacterial 16S rRNA gene were amplified with primers F515/R907 [50] and sequencing was performed on an Illumina Miseq PE300 platform (Illumina Inc.). More details for amplicon sequencing are included in the Supporting Information: Methods. Raw fastq files were demultiplexed and quality‐filtered using the QIIME 2 pipeline [51]. Adapters were trimmed with the qiime cutadapt trim‐paired tool. Paired reads were joined (minimum overlapping read length of 20 base pairs) and quality filtered (Phred score of 20), and reads with less than 200 base pairs were removed. Chimeras were screened and removed using the UCHIME algorithm [52]. Sequences were then assigned to OTU at a 97% similarity level with UPARSE [53]. A representative sequence of each OTU was taxonomically classified with the SILVA database (v132) [54]. One specific OTU affiliated with *Pseudomonas* sp., OTU1633, significantly responded to biochar amendment (see “Results” section for more details). Sequences belonging to chloroplasts, mitochondria, and archaea were removed. To avoid potential bias caused by sequencing depth, the number of sequences was rarefied to the minimum number of sequences (32,352 sequences) per sample using the “vegan” package [55] in “R” (v4.1.0, http://www.r-project.org/).

The abundance of *Pseudomonas* sp. was assessed by SYBR Green quantitative PCR assays with primers Pse435F/Pse686R [56], which specifically targets the 16S rRNA gene of this genus. The abundance of FOL was assessed by TaqMan quantitative PCR assays with FOL3f/FOL3r and probe TaqMan3 [57], which targets the virulence gene *SIX1* of FOL. More details for quantitative PCR are included in the Supporting Information: Methods.

### Isolation and characterization of culturable bacteria

Culturable bacteria were isolated from tomato rhizosphere soils of the biochar treatment taken from the pot experiment using the plate culturing method as previously described [58]. These isolates were taxonomically classified using 16S rRNA gene sequencing with primers 27F/1492R [59], and we obtained 29 *Pseudomonas* sp. isolates. After the elimination of potential clonal duplicates, that is, isolates with 100% identity of the 16S rRNA gene sequence, we obtained 10 *Pseudomonas* sp. isolates. From these isolates, we selected one isolate (TP27), which displayed 100% sequence similarity with OTU1633. The in vitro antagonistic activities of TP27 against FOL were evaluated using the dual culture test on the PDA plate as previously described [38]. Then, a greenhouse experiment was performed to assess the effects of TP27 on tomato seedling growth, *Fusarium* wilt disease severity. Expressions of defense‐related genes, including *AOS* and *PAL* genes, in tomato roots were also determined. To assess if TP27 could induce systemic responses in tomato plants, we monitored *AOS* and *PAL* genes, which are involved in the jasmonic acid and salicylic acid signaling pathways, respectively [41, 42]. Briefly, total RNA was isolated from tomato root samples using the TRIZOL reagent (Invitrogen), and the first‐strand complementary DNA was synthesized with the oligo (dT)_15_ primer using the TIANScript RT Kit (Tiangen Biotech). All primers are provided in the Supporting Information: Methods. Quantitative PCR assays were performed on an IQ5 Real‐Time PCR System (Bio‐Rad Lab). The relative expression of these genes was calculated with the $2^{-\Delta \Delta C_{T}}$ method [60]. Details of these methods are described in Supporting Information: Methods.

### Microcosm experiment

A microcosm was performed to assess the effect of biochar amendment on *Pseudomonas* sp. TP27 growth in autoclaved soil. Briefly, autoclaved cropland soils were inoculated with TP27 suspension (1.0 × 10^7^ colony‐forming unit [CFU ml^−1^]). Three days later, these soils were (i) not amended with biochar (the control), or (ii) amended with biochar at the rate of 1% (w/w). Then, jars containing different soils were incubated at 28°C in the dark with soil water content maintained at 60% of WHC. There were three replicates for each treatment and five jars per replicate. The cell numbers of TP27 in the soil were measured on 7, 14, and 21 days after incubation with the plate counting method on LB agar.

### Split‐root system to assess the colonization of *Pseudomonas* sp. TP27 on tomato roots

A split‐root system of tomato was performed to assess the effect of biochar amendment on the colonization of *Pseudomonas* sp. TP27 on tomato roots. The advantage of the split‐root system was that it could spatially separate TP27 from biochar. A spontaneous rifampicin‐resistant mutant of TP27 was generated as previously described [61, 62]. Cropland soils were sterilized as described above, and one portion of these soils was amended with biochar at the rate of 1% (w/w), another portion was inoculated with a suspension of TP27 (1.0 × 10^7^ CFU g^−1^ soil, the rifampicin‐resistant mutant), while the other was not amended or inoculated. When the radicle of germinated tomato seeds had emerged, the distal 1 mm of root tip was removed and the seedlings were cultivated in sterile soil. About 4 weeks later, the root system of tomato seedlings was divided into two equal parts and placed in two separate adjacent pots (10 cm × 10 cm, containing 700 g of soils), such that the two separate parts of the root system were in separate pots [3]. There were two treatments: (i) one part of tomato seedling root was grown in soils amended with biochar, while the other part of the root in soils inoculated with TP27, and (ii) one part of tomato seedling root was grown in soils nonamended with biochar, while the other part of the root in soils inoculated with TP27 (the control). Each treatment was replicated three times, and each replicate contained 10 pots. Seedlings were maintained as described above.

Fifteen days later, five tomato seedlings of each treatment in each replicate were harvested to measure dry biomass and collect rhizosphere soils from tomato roots in the pots that were inoculated with TP27. The cell number of TP27 was counted by plating on LB agar amended with 150 μg ml^−1^ rifampicin after incubated at 28°C in the dark for 2–3 days.

Meanwhile, five tomato seedlings of each treatment in each replicate were harvested to collect root exudates (Supporting Information Methods). Effects of exudates from roots in soil inoculated with TP2 on the chemotaxis and biofilm formation of *Pseudomonas* sp. TP27 were analyzed in vitro using the microtiter plate assay and modified capillary assay methods, respectively, as previously described [47].

### Statistical analyses

All data were checked for normality (Shapiro–Wilk's test) and homogeneity of variances (Levene's test). Data of microbial abundances as measured by quantitative PCR and plate counting were logarithmically transformed. A comparison between two groups was performed using Welch's *t *test. For more than two groups, a one‐way analysis of variance, followed by Tukey's HSD test was performed to determine the statistical significance between treatments. For the amplicon sequencing data, bacterial community α‐diversities were calculated as the number of OTUs and Shannon index using the “vegan” package [55]. The β‐diversity was analyzed using PCoA based on the Bray–Curtis dissimilarities at the OTU level. Differences in the relative abundance of OTUs between treatments were measured using likelihood ratio tests with the Benjamini–Hochberg *p* value correction in the “EdgeR” package [63].

## AUTHOR CONTRIBUTIONS

Xue Jin, Xiaojun Kang, Kai Pan, Fengzhi Wu, and Xingang Zhou conceived and designed the study. Xue Jin, Yang Bai, Muhammad Khashi u Rahman, and Xingang Zhou performed the experiments and analyzed the data. Xue Jin, Muhammad Khashi u Rahman, Thomas Pommier, Xingang Zhou, and Zhong Wei wrote the manuscript. All authors edited the manuscript and approved the final draft.

## CONFLICT OF INTEREST

The authors declare no conflict of interest.

## Supporting information

Supplementary information.

## Data Availability

The raw sequencing data were deposited in the Sequence Read Archive at NCBI with the submission accession number PRJNA797541 (https://www.ncbi.nlm.nih.gov/bioproject/PRJNA797541). Supporting Information: Materials (figures, tables, scripts, graphical abstract, slides, videos, Chinese translated version and updated materials) may be found in the online DOI or iMeta Science http://www.imeta.science/.
