# Supplementary material for: Biochar stimulates tomato roots to recruit a bacterial assemblage contributing to disease resistance against Fusarium wilt
Source: Imeta. 2022 Jun 23;1(3):e37. doi: 10.1002/imt2.37 (PMC10989760; doi:10.1002/imt2.37)
Supplement: Supplementary file 1 — Supplementary information. [file IMT2-1-e37-s001.docx]

Supporting Information for

**Biochar stimulates tomato roots to recruit a bacterial assemblage contributing to disease resistance against Fusarium wilt**

**Supplementary Methods**

**Collection of tomato seedling rhizosphere soils**

Tomato seedling rhizosphere soils were collected as previously described [[1](#_ENREF_1)]. Briefly, tomato roots were carefully taken out of the pots, and soils loosely attached to the roots were removed by manual shaking. Then, soils tightly adhering to the roots were removed from the root surface with a sterile brush and considered as rhizosphere soils [[2](#_ENREF_2)]. Samples from 20 plants of each treatment in each replicate were combined to make a composite sample. Finally, these soil samples were sieved (2 mm).

**Quantitative PCR conditions for *Pseudomonas* sp. and FOL**

Quantitative PCR assays were conducted with an IQ5 real-time PCR system (Bio-Rad Lab, LA, USA). For *Pseudomonas* sp., quantitative PCR assays were performed in a 25 μl reaction mixture containing 12.5 μl of 2×Real SYBR Mixture (Tiangen Biotech, Beijing, China), 0.2 μM of each of the forward and reverse primers, 5 ng of DNA, and sterile deionized water was used to bring the total volume to 25 μl. The PCR protocols were 94°C for 5 min; followed by 94°C for 45 s, 56°C for 30 s, 72°C for 45 s, 30 cycles; and a final extension at 72°C for 10 min.

For FOL, quantitative PCR assays were performed in a 25 μl reaction mixture containing 12.5 μl of FastFire TaqMan qPCR PreMix (Tiangen Biotech, Beijing, China), 0.2 μM of each of the forward and reverse primers, 0.15 μM of dual labeled TaqMan probes with 5′-6-FAM fluorescent dye and 3′-BHQ-1 quencher, 5 ng of DNA, and sterile deionized water was used to bring the total volume to 25 μl. The PCR protocols were 94°C for 5 min; followed by 94°C for 30 s, 60°C for 20 s; 40 cycles. Fluorescence emission was measured at 60°C during the annealing and extension phase.

Standard curves were created with 10-fold dilution series of plasmids containing the target gene of an isolated *Pseudomonas* sp. TP27 from tomato rhizosphere or FOL. The threshold cycle values obtained for each sample were compared with the standard curve to calculate the copy number of the target gene. The efficiency of the reaction was between 97% and 100% (based on the slopes of the standard curves). Sterile deionized water was used as a negative control to replace the template. All amplifications were performed in triplicate.

**Amplicon sequencing of bacterial 16S rDNA**

The V4−V5 regions of the bacterial 16S rDNA were amplified with primers F515/R907 [[3](#_ENREF_3)] with specific overhang Illumina adapters. PCR was performed in a reaction mixture of 25 μl consisting of 12.5 μl of 2×Taq PCR MasterMix (Tiangen Biotech, Beijing, China), 0.2 μM of each of the forward and reverse primers, 5 ng of DNA; sterile deionized water was used to bring the total volume to 25 μl. The PCR was performed with an EasyCycler PCR System (Analytik Jena, Jena, Germany) using a program of 95°C for 5 min; followed by 35 cycles of 95°C for 30 s, 55°C for 30 s, 72°C for 45 s; and a final extension at 72°C for 10 min. To avoid DNA contaminations originating from kits and reagents, sterile deionized water was used as a negative control. There were three technically replicated PCR reactions for each soil DNA sample. The products of three technically replicated PCR reactions were pooled and purified. A second eight-cycle PCR was performed to add dual index and Illumina sequencing adapters using a Nextera XT Index Kit (Illumina Inc., San Diego, USA). Then, PCR products were purified, quantified and normalized prior to pooling. Finally, the DNA library pool was paired-end sequenced (2×300) on an Illumina Miseq PE300 platform (Illumina Inc., San Diego, USA).

**Isolation and identification of culturable *Pseudomonas* sp.**

Culturable bacteria were isolated from tomato rhizosphere soils taken from the pot experiment. For isolation of *Pseudomonas* sp. from tomato rhizosphere soils, one gram of soil was placed into a 50 ml flask containing 10 ml of a 0.9% NaCl solution, and the flask was shaken for 30 min at 400 rpm. Then, the suspension was serially diluted and plated on the selective medium Gould’s S1 [[4](#_ENREF_4)]. After incubation at 28 °C for 48 h, colonies were selected and purified on Luria-Bertani agar.

Genomic DNA was extracted from liquid cultures of bacteria isolates using the TIANamp Bacteria DNA Kit (Tiangen Biotech, Beijing, China) following the manufacturer's protocols. Bacterial 16S rRNA genes were amplified with primers 27F/1492R [[5](#_ENREF_5)]. PCR assays were performed in a 25 μl reaction mixture containing 12.5 μl of 2×Taq PCR MasterMix (Tiangen Biotech, Beijing, China), 0.2 μM of each of the forward and reverse primers, 2.5 ng of DNA, and sterile deionized water was used to bring the total volume to 25 μl. The PCR protocols were 94°C for 3 min; followed by 94°C for 30 s, 58°C for 45 s, 72°C for 90 s, 30 cycles; and a final extension at 72°C for 5 min. The amplicons were purified and commercially sequenced by the Sangon Biotech Co., Ltd., Shanghai, China. Then, the obtained sequences were compared to other sequences on the BLAST database at the National Center for Biotechnology Information (https://blast.ncbi.nlm.nih.gov/Blast.cgi). Phylogenetic analyses were conducted using MEGA (version 10.1.7) [[6](#_ENREF_6)] and the neighbor-joining tree was constructed using Kimura 2-parameter distance with 1,000 replicates to produce Bootstrap values. The 16S rRNA gene sequences of *Pseudomonas* sp. isolates were matched with OTU1633. The sequences of bacterial 16S rRNA gene were trimmed at the sites of primer sets F515/R907. Then, the sequence similarities of each *Pseudomonas* sp. isolates with OTU1633 were determined using Blast.

**Testing the ability of *Pseudomonas* sp. TP27 to suppress Fusarium wilt disease**

A pot experiment with sterilized cropland soils was performed to evaluate the efficacy of *Pseudomonas* sp. TP27 to control tomato Fusarium wilt disease. Briefly, tomato seedlings with two cotyledons were transplanted in plastic pots (16 cm × 14 cm) containing 1 kg of sterilized cropland soils. Overnight LB broth culture of TP27 was pelleted by centrifugation (5000 *g*, 5 min), washed three times and re-suspended in phosphate buffer saline (PBS) solution (8.0 mg ml^−1^ NaCl, 0.2 mg ml^−1^ KCl, 1.44 mg ml^−1^ Na_2_HPO_4_, 0.24 mg ml^−1^ KH_2_PO_4_, pH 7.2) at a final cell density of 10^7^ CFU ml^−1^. Two weeks after transplantation, tomato seedlings were inoculated with *Pseudomonas* sp. TP27 and FOL06 of FOL. There were four treatments: (i) non-inoculated control; (ii) inoculation only with FOL06; (iii) inoculation only with TP27; (iv) inoculation with both FOL06 and TP27. Ten milliliters of TP27 suspension (1.0×10^7^ CFU ml^−1^) was applied as a soil drench in each pot. Five days after the introduction of TP27, 10 ml of conidial suspension of FOL06 (1.0×10^7^ conidia ml^−1^) was directly pipetted onto the soil surface of each pot. Each treatment was replicated three times with 15 pots in each replicate. All pots were placed randomly in the greenhouse (average day/night temperature 32℃/22℃, relative humidity 60-80%, 16 h light). Soil water content was adjusted every two days with distilled water to maintain the soil moisture at about 60% of the water holding capacity.

Tomato seedling root samples were harvested at 0, 12 and 24 hours post-inoculation of FOL06. Samples from five seedlings of each treatment in each replicate were combined to make a composite sample. After sampling, samples were immediately frozen in liquid nitrogen and then stored at −80°C. Two weeks after inoculation of FOL06, Fusarium disease severity and seedling dry biomass were evaluated as described above.

**Quantification of defense-related gene expressions**

Total RNA was isolated from tomato root samples using the TRIZOL reagent (Invitrogen, Carlsbad, USA) according to the manufacturer’s protocols. The yield and quality of total RNA were checked with 1.2% (w/v) agarose gel electrophoresis and a NanoDrop 2000 spectrophotometer (ThermoFisher Scientific, Wilmington, USA). Afterward, the first-strand cDNA was synthesized with the primer oligo (dT)_15_ using the TIANScript RT Kit (Tiangen Biotech, Beijing, China) according to the manufacturer’s protocols. The expressions of defense-related genes, including genes encoding pathogenesis-related protein 1 (*PR1a*) and phenylalanine ammonia-lyase (*PAL*), were analyzed using SYBR-Green based quantitative reverse transcription-PCR (qRT-PCR) on an IQ5 real-time PCR system (Bio-Rad Lab, Hercules, USA). The *ACTIN* gene was used as the reference gene. The following primers were used: *AOS* gene primers (forward, 5'-TCT CTT CCT CTT CCT TCT CTT CAC C-3'; reverse, 5'-CGC CGG GTA TAG TCC TGG TAG ATA-3'), *PAL* gene primers (forward, 5'-CGT TAT GCT CTC CGA ACA TC-3'; reverse, 5'-GAA GTT GCC ACC ATG TAA GG-3'), *ACTIN* gene primers (forward, 5'-TGA ATG CAC GGT AGC AAA CAA CAG ATT-3'; reverse, 5'-AAT GCA TCA GGC ACC TCT CAA GTA T-3') [[7-9](#_ENREF_7)]. Quantitative PCR assays were performed in a 25 μl reaction mixture containing 12.5 μl of 2×Real SYBR Mixture (Tiangen Biotech, Beijing, China), 0.2 μM of each of the forward and reverse primers, 2 μl of cDNA, and sterile deionized water was used to bring the total volume to 25 μl. The PCR protocols were 95°C for 1 min; followed by 95°C for 15 s, 58°C for 15 s, 72°C for 30 s, 26 cycles; and a final extension at 72°C for 5 min. Sterile deionized water was used as a negative control to replace the template. All amplifications were performed in triplicate. The specificity of the products was confirmed by melting curve analysis. Relative expression of these genes was calculated with the 2^-ΔΔCT^ method [[10](#_ENREF_10)].

**Collection of tomato seedling root exudates**

Tomato seedling root exudates were collected as previously described [[11](#_ENREF_11),[12](#_ENREF_12)]. Plant seedlings were carefully removed from the pots and roots were washed with sterile deionized water. Then, tomato seedlings from each treatment in each replicate were put into two beakers with the root systems inoculated with TP27 in one baker and the other root systems in another baker. Each baker contained 200 ml of sterile deionized water containing Calcium chloride (0.5 mM) to maintain the root membrane integrity [[13](#_ENREF_13)]. The beaker was wrapped with tin paper to prevent light and placed in a growth chamber at 28℃ under light for 6 h. Then, the roots were dried with Whatman's No. 1 filter paper (Whatman Lab Sales Ltd., Maidstone, UK) and weighed. The concentration of the solution was adjusted with sterile deionized water to make a final concentration of 1 g fresh weight of root per 10 ml exudate solution. Finally, these solutions were filtered through 0.22 μm membranes (Merck, Darmstadt, Germany).

***In vitro* biofilm and chemotaxis assays**

The biofilm formation was determined using a microtiter plate assay. Briefly, overnight LB broth culture of TP27 was pelleted by centrifugation (5000 *g*, 5 min), washed three times and re-suspended in 1/10 LB broth medium. Each well of 48-well microtiter plates was filled with 1 ml of freshly prepared 1/10 LB broth medium, and inoculated with 10 μl of TP27 suspension. Then, 20 μl of root exudate or 1/10 LB broth medium (negative control) were added. Each treatment was replicated three times with five wells for each replicate. After static incubation at 27°C for 16 h, growth medium and non-adherent cells were removed from the microtitre plate wells. Cells that had adhered to the wells were stained with 1 ml of 0.1 % crystal violet for 30 min at room temperature. Biofilm formation was quantified by measuring the OD_600_ for each well using a Bio-Tek Epoch ELX800 microplate spectrophotometer (Winooski, VT, USA).

The chemotaxis of TP27 to tomato root exudates was determined using a modified capillary assay. Briefly, 200-ml pipette tip was filled with 100 ml of TP27 suspension (OD_600_ = 0.1), which were prepared from an overnight LB broth culture. A 4-cm 25-gauge needle was used as the chemotaxis capillary and was attached to a 1-ml tuberculin syringe containing 200 ml of root exudates. The tip of the needle syringe was connected to bacterial suspension in the pipette tip. After static incubation at 27°C for 16 h, the needle syringe was removed from the bacterial suspension and the cell number of TP27 in the syringe was counted by plating on LB agar medium. Each treatment was replicated three times with five wells for each replicate.

**Supplementary Figures**

**
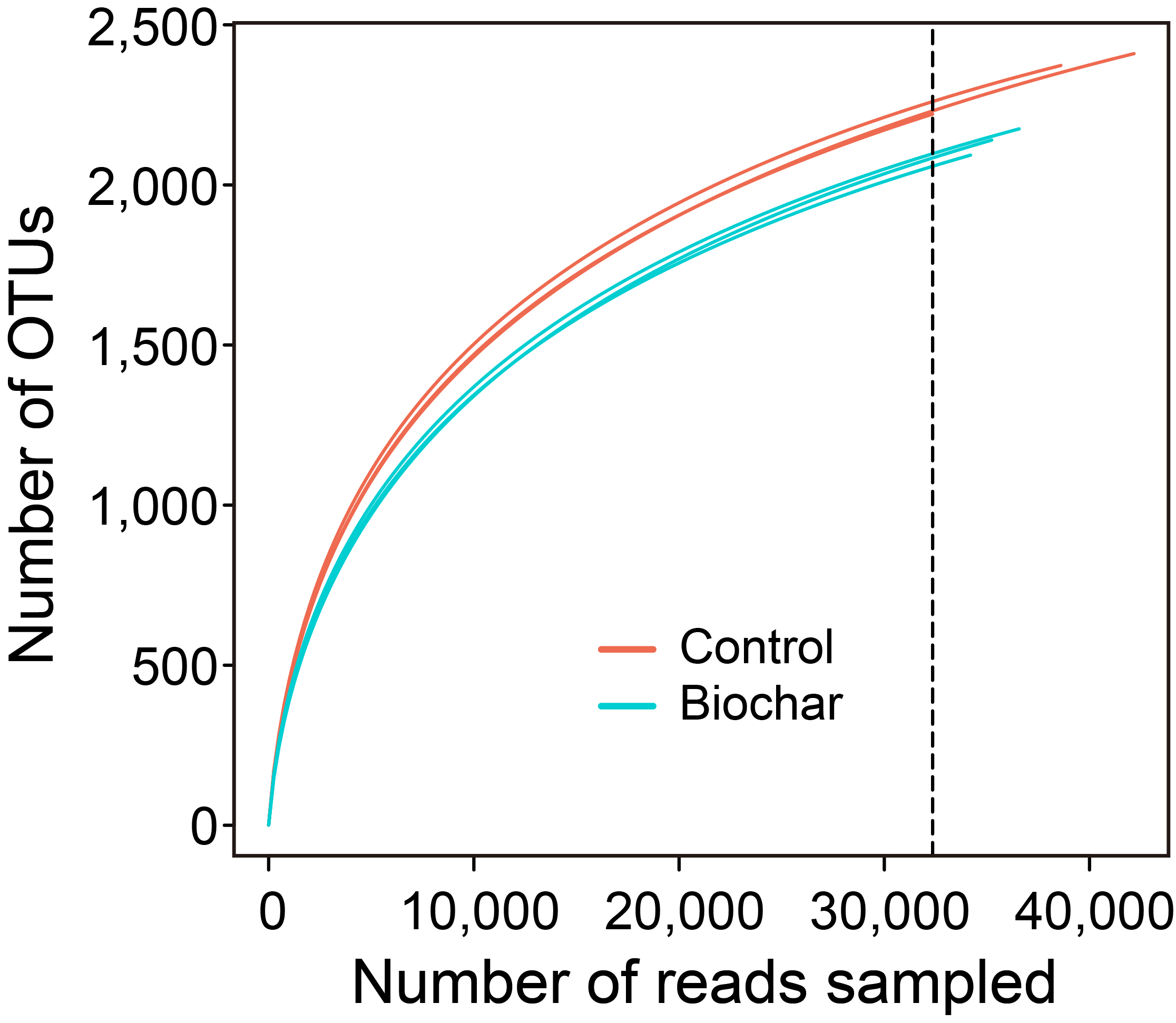
**

**Fig. S1** Rarefaction curves of the number of OTUs at the 97% sequence similarity. The dashed black line indicates the selected rarefaction depth (32,352 sequences per sample) used to calculate the alpha diversity of the bacterial community in tomato seedling rhizosphere.


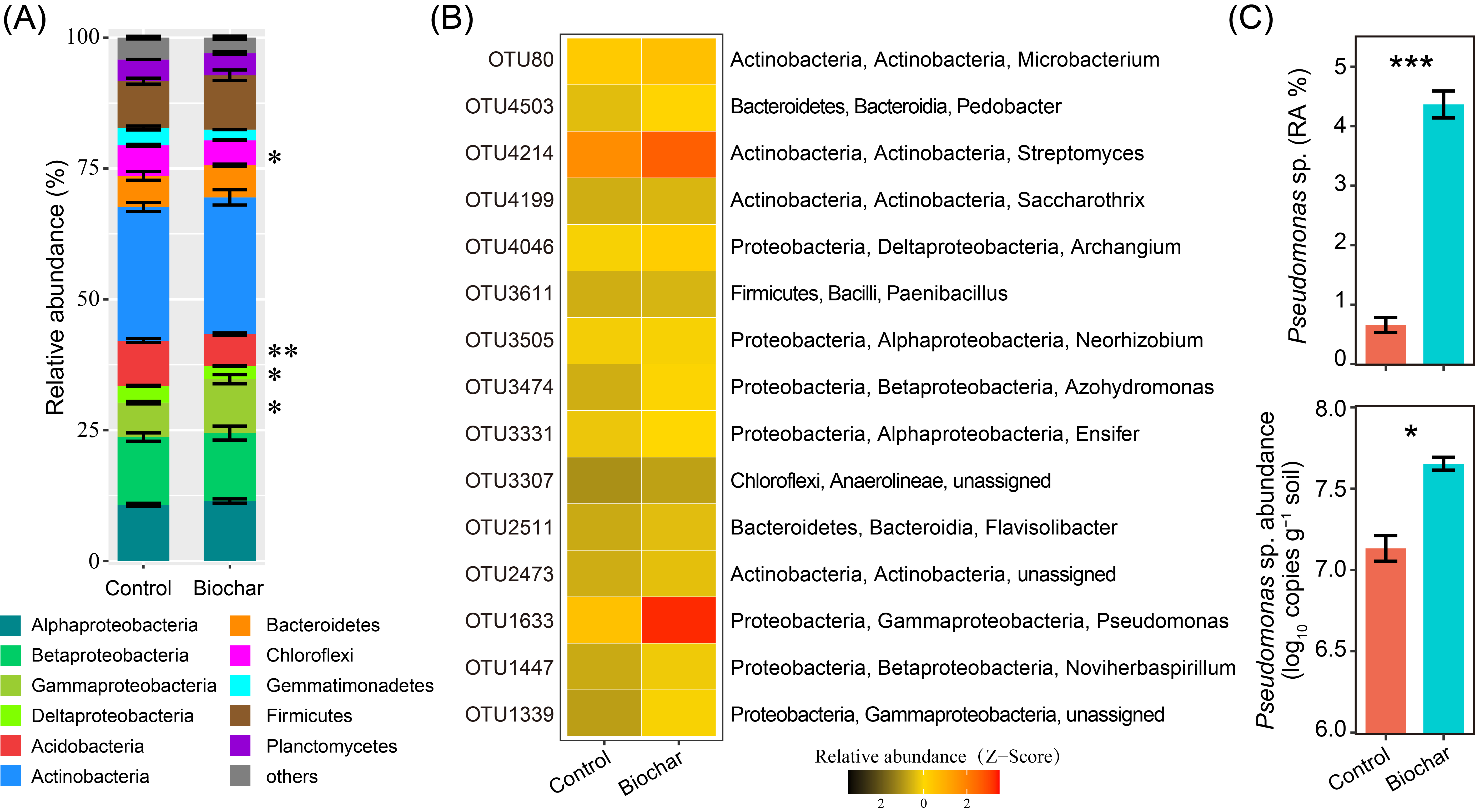


**Fig. S2** Changes in the taxonomic composition of bacterial community in tomato rhizosphere. **(a)** Relative abundances of main bacterial phyla/Proteobacteria classes (mean relative abundances > 1%) of each treatment. Each bar represents the mean value of three replicates. **(b)** The heatmap showing the relative abundances of bacterial OTUs stimulated by the biochar amendment. The taxonomic information of each OTU at the phylum, class and genus was shown. **(c)** The relative abundance of *Pseudomonas* sp. (estimated by 16s rRNA gene sequencing) and the abundance of *Pseudomonas* sp. (estimated by quantitative PCR) in tomato rhizosphere. Values are represented as mean ± SE (*n*=3). *, **, and *** indicate significant difference at *P* < 0.05, *P* < 0.01 and *P* < 0.001 (Welch’s *t*-test), respectively.


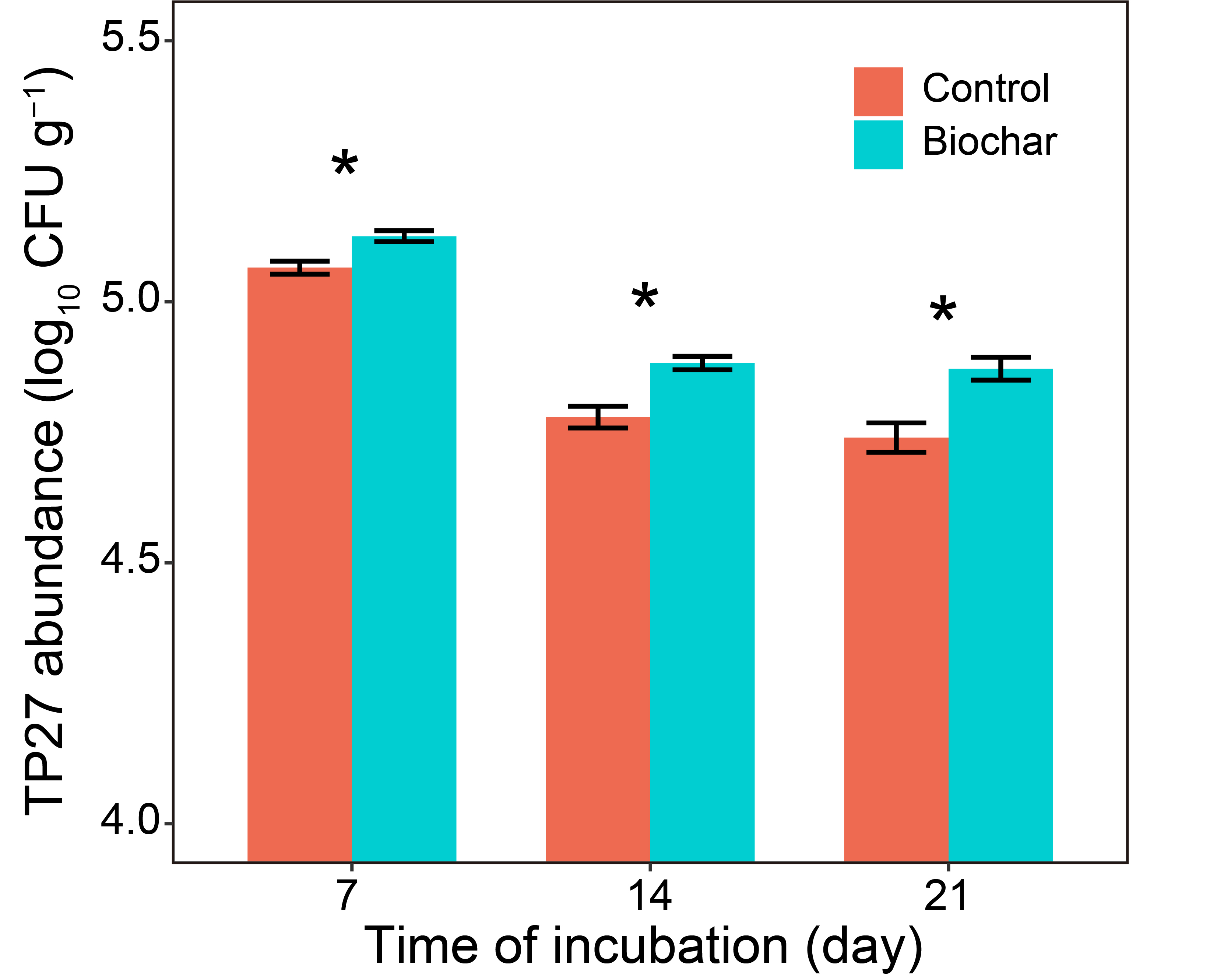


**Fig. S3** Effects of biochar on *Pseudomonas* sp. TP27 abundance in the soil. Values are represented as mean ± SE (*n*=5). * indicates significant difference at *P* < 0.05 (Welch’s *t*-test).

**Supplementary references**

1. Jin, Xue, Wu Fengzhi, and Zhou Xingang. 2020. "Different toxic effects of ferulic and *p*-hydroxybenzoic acids on cucumber seedling growth were related to their different influences on rhizosphere microbial composition." *Biology and Fertility of Soils* 56: 125-136. doi: 10.1007/s00374-019-01408-0

2. Chen, Yan, Bonkowski Michael, Shen Yi, Griffiths Bryan S., Jiang Yuji, Wang Xiaoyue, and Sun Bo. 2020. "Root ethylene mediates rhizosphere microbial community reconstruction when chemically detecting cyanide produced by neighbouring plants." *Microbiome* 8: 4. doi: 10.1186/s40168-019-0775-6

3. Zhou, Jizhong, Wu Liyou, Deng Ye, Zhi Xiaoyang, Jiang Yi-Huei, Tu Qichao, Xie Jianping, et al. 2011. "Reproducibility and quantitation of amplicon sequencing-based detection." *ISME Journal* 5: 1303-1313. doi: 10.1038/ismej.2011.11

4. Gould, W.D, Hagedorn C.., Bardinelli T.R., and Zablotowicz R.M. 1985. "New selective media for enumeration and recovery of fluorescent *Pseudomonads* from various habitats." *Applied and Environmental Microbiology* 49: 28-32

5. Heuer, H., Krsek M., Baker P., Smalla K., and Wellington E. M. 1997. "Analysis of actinomycete communities by specific amplification of genes encoding 16S rRNA and gel-electrophoretic separation in denaturing gradients." *Applied and Environmental Microbiology* 63: 3233-3241. doi: 10.1128/aem.63.8.3233-3241.1997

6. Tamura, Koichiro, Peterson Daniel, Peterson Nicholas, Stecher Glen, Nei Masatoshi, and Kumar Sudhir. 2011. "MEGA5: molecular evolutionary genetics analysis using maximum likelihood, evolutionary distance, and maximum parsimony methods." *Molecular Biology and Evolution* 28: 2731-2739. doi: 10.1093/molbev/msr121

7. Casteel, Clare L., Hansen Allison K., Walling Linda L., and Paine Timothy D. 2012. "Manipulation of plant defense responses by the tomato psyllid (*Bactericerca cockerelli*) and its associated endosymbiont *Candidatus* Liberibacter psyllaurous." *Plos One* 7: e35191. doi: 10.1371/journal.pone.0035191

8. Chandrasekaran, Murugesan, Belachew Shimels Tilahun, Yoon Ee, and Chun Se Chul. 2017. "Expression of beta-1,3-glucanase (*GLU*) and phenylalanine ammonia-lyase (*PAL*) genes and their enzymes in tomato plants induced after treatment with *Bacillus subtilis* CBR05 against *Xanthomonas campestris* pv. *vesicatoria*." *Journal of General Plant Pahtology* 83: 7-13. doi: 10.1007/s10327-016-0692-5

9. Di, Xiaotang, Gomila Jo, and Takken Frank L. W. 2017. "Involvement of salicylic acid, ethylene and jasmonic acid signalling pathways in the susceptibility of tomato to *Fusarium oxysporum*." *Molecular Plant Pathology* 18: 1024-1035. doi: 10.1111/mpp.12559

10. Livak, Kenneth J, and Schmittgen Thomas D. 2001. "Analysis of relative gene expression data using real-time quantitative PCR and the 2^−ΔΔCT^ method." *Methods* 25: 402-408. doi: 10.1006/meth.2001.1262

11. Hao, Wen-ya, Ren Li-xuan, Ran Wei, and Shen Qi-rong. 2010. "Allelopathic effects of root exudates from watermelon and rice plants on *Fusarium oxysporum* f.sp. *niveum*." *Plant Soil* 336: 485-497. doi: 10.1007/s11104-010-0505-0

12. Neal, Andrew L, Ahmad Shakoor, Gordon-Weeks Ruth, and Ton Jurriaan. 2012. "Benzoxazinoids in root exudates of maize attract *Pseudomonas putida* to the rhizosphere." *PloS One* 7: e35498. doi: 10.1371/journal.pone.0035498

13. Vranova, Valerie, Rejsek Klement, Skene Keith R., Janous Dalibor, and Formanek Pavel. 2013. "Methods of collection of plant root exudates in relation to plant metabolism and purpose: A review." *Journal of Plant Nutrition and Soil Science* 176: 175-199. doi: 10.1002/jpln.201000360
